# The effectiveness of community friendship groups on participant social and mental health: a meta-analysis

**DOI:** 10.3389/fpsyg.2023.1078268

**Published:** 2023-12-07

**Authors:** Micah Grishina, Rosanna Mary Rooney, Lynne Millar, Rachel Mann, Vincent Oreste Mancini

**Affiliations:** ^1^Discipline of Psychology, School of Population Health, Curtin University, Perth, WA, Australia; ^2^Telethon Kids Institute, Perth, WA, Australia; ^3^Division of Paediatrics, UWA Medical School, University of Western Australia, Perth, WA, Australia; ^4^The Fathering Project, Sydney, NSW, Australia

**Keywords:** community friendship groups, Social Isolation, social capital, social connectedness, depression, social and mental health, social support

## Abstract

**Introduction:**

Social disconnectedness and isolation are risk factors for poor mental health. Community-based friendship group interventions have been designed to increase an individual’s social capital and consequently their mental wellbeing. Structured and unstructured friendship groups reflect two distinct approaches to friendship group interventions.

**Methods:**

This meta-analysis investigated whether structured or unstructured community friendship groups are more effective for mental health and social capital outcomes. A systematic search of quantitative studies was conducted across seven databases and study quality was assessed using the Effective Public Health Practice Project (EPHPP) tool. Eight studies (2 unstructured and 6 structured friendship groups) were included in the review, published between 2005 and 2020.

**Results:**

Structured friendship groups had a small significant effect on reducing participant depression symptoms. There was not enough available data to compare the effectiveness of structured and unstructured groups for mental health outcomes. There was substantial heterogeneity between studies to calculate pooled effect sizes for any social capital outcomes. Data synthesis indicated mixed reviews for social capital outcomes, likely due to the large heterogeneity and limited studies.

**Discussion:**

This meta-analysis provides limited support for positive mental health outcomes following structured community-based friendship group interventions. There is a need for additional research as a large research gap remains, particularly for unstructured friendship groups.

**Systematic Review Registration:**

https://www.crd.york.ac.uk/prospero/display_record.php?RecordID=260639, CRD42021260639.

## Introduction

The growing economic and community burden of mental health disorders has been increasingly recognized as a priority for governments, both domestically and internationally (Coffey and Hannigan, 2005). Mental health disorders can be defined as changes in an individual’s emotion, cognition, and behavior that result in significant dysfunction to areas of the individual’s life, such as their social or occupational functional ability (American Psychiatric Association, 2013). Examples of mental health disorders include depression, anxiety, schizophrenia, and dementia (American Psychiatric Association, 2013). Depressive disorders were found to be amongst the leading cause of global burden of disease and disability in 2017 (James et al., 2018). Additionally, ongoing COVID-19 restrictions and consequences have had a considerable impact on increasing the overall psychological distress, and the prevalence and functional impact of mental health disorders (see Kola et al., 2021).

Particularly with the ongoing global impact of COVID-19 restrictions and uncertainty, social disconnectedness and isolation remain a risk factor for declining mental health among both clinical and non-clinical groups (Saeri et al., 2018; Kola et al., 2021). Disconnectedness refers to an individual’s separation from other individuals and implies a separation from social connection. An individual can perceive themself as disconnected from others and socially isolated when in fact the individual is physically nearby other individuals who may provide social connectedness (Cornwell and Waite, 2009). This perception, or reality, can underlie an individual’s negative self-concept (Quach and Burr, 2020). Disconnectedness and social isolation, whether perceived or indeed true for an individual, encompass some of the biggest barriers faced by individuals experiencing a mental health disorder or general low mental wellbeing (Hämmig, 2019). Forming and maintaining friendships where an individual perceives social connectedness can therefore often be crucial to an individual’s well-being.

A widely investigated intervention target has been identified as increasing individual’s social interactions and perceived connectedness and support through friendships (see Cohen, 2004). This is often referred to as *social capital* (SC). According to Putnam (2000), social capital refers to an individual’s perceived quality of their social relationships and the nature of their participation to surrounding community networks. Part of increasing an individual’s social capital, is impacted by their social identification (Tajfel and Turner, 1986). In line with Social Identification Theory (SIT; Tajfel and Turner, 1986), Haslam et al. (2009) recognized that social identification with a social group provides an adjacent sense of self in the face of change and uncertainty. This suggests that friendships can become a source of social support for an individual experiencing adversity. When a loss of an old identity occurs, group memberships deemed as positive influences on an individual’s social capital can strengthen opportunities to develop social connectedness (friendships). These social identities are said to endure benefits to that individual’s health (Haslam et al., 2016). Jetten et al. (2011) named this as a “social cure” that impacts mental health by promoting adjustment and coping and contributing to overall well-being.

The accessibility of social capital is important to investigate given the ongoing unpredictability of adverse events, community and family displacement, isolation, and immigration (Navarra et al., 2013; Aldrich and Meyer, 2015). Research has investigated various means of implementing interventions that target an increase in social capital by facilitating friendships and social relationships to increase mental health wellbeing. Poscia et al.’s (2018) systematic review investigated interventions targeting loneliness and social isolation amongst older adults. The review found evidence for promising strategies to decrease social isolation and loneliness, using various technology and community-based interventions. In particular, community groups have received optimistic attention due to their degree of flexible implementation, cost-effectiveness, and they can work to influence multiple individuals at once across various social groups (Kazdin, 2019). Group settings are supported as particularly accessible providers of opportunities of social support, social skills development, and the integration of individuals into a community (Drake and Whitley, 2014; Meiring et al., 2017).

Community-based group interventions can be delivered in structured or unstructured ways (Leeman et al., 2015). Structured interventions typically involve the delivery of an intervention in a particular manner, such as manualized intervention programs. For example, Haslam et al. (2016) investigated the effectiveness of a manualized psychological intervention (Groups 4 Health) targeting social isolation and associated psychological distress in the community. Unstructured community-based group interventions may include the provision of an informal space for individuals to gather and engage in a range of unstructured and often unplanned activities or networking. Meiring et al. (2017) investigated the effectiveness of a student-led support group initiative for mental health care users in a South African community health center. The social support was an unstructured group that offered socially isolated and stigmatized users of the community health center. Previous literature has also investigated the use of community based and informal social support interventions for individuals diagnosed with a serious mental illness (e.g., Corrigan et al., 2002; McCorkle et al., 2009). Unstructured groups may hold greater cost-effectiveness compared to structured groups, due to the informal nature of the group often requiring less training for group facilitators and cost of material (e.g., booklets) used (Jetten et al., 2014). Structured groups may be too formal for individuals wishing to expand their social capital or for individuals who may not want formal skills training in making friends (e.g., Haslam et al., 2019). Given the importance of social capital in promoting mental health wellbeing outcomes, there is limited knowledge regarding the type of community groups that best improve an individual’s social capital and mental health.

The impact of social capital and mental health has been previously investigated through a systematic review of social capital interventions and their impact on mental health (Flores et al., 2018). Whilst the review indicated various social capital interventions existed and had positive short-term impacts on mental health and the social capital of individuals, the review indicated that heterogeneity exists in current research regarding social capital definitions and mental health outcomes. Further, Flores et al. (2018) did not investigate the *type* of friendship group nor the specific focus on friendship as the main social capital-based intervention despite the established importance of social connection and identification. Lastly, Flores et al. (2018) found minimal long-term differences between intervention and control groups for social capital and mental health outcomes. There remains a need to investigate the specific comparison of social capital and mental health outcomes between structured versus unstructured community-based social (friendship) groups (and any long-term effects) to allow for the addition of interventions evaluated and published since Flores et al.’s (2018) systematic review.

A debate exists in literature regarding the homogeneity of social capital-based definitions that exist and guide intervention-based research. De Silva et al. (2005) review on social capital and mental illness highlighted methodological limitations; varying social capital definitions may not be completely captured in systematic searches, despite likely meeting inclusion criteria for social support interventions. As such, it would be important to capture a broader review of available literature for community-based social support interventions and mental health impacts. This systematic-review and meta-analysis will also be conducted to address the discussed limitations of prior research and capture a broader and updated review of friendship group and social capital literature.

For the purposes of this meta-analysis, a community friendship group is defined as an unstructured or structured group provided by paid or volunteer members of a local government council or organization, to facilitate individuals to meet at a certain place and time and socially interact. The intention of using the framework of ‘friendships groups’ as opposed to social capital or social support groups is to account for reported methodological difficulties noted by De Silva et al. (2005). The aim of the proposed meta-analysis is to provide an updated review of available structured and unstructured community-based friendship group interventions that target social capital-based concepts (e.g., perceived social support and belongingness) and the outcomes of these on mental health. This study will also aim to extend Flores et al.’s (2018) systematic review by conducting a meta-analysis on the outcomes. The following hypotheses will be addressed:

1. It is hypothesized that structured community friendship groups will show greater effectiveness for mental health outcomes in participants compared to unstructured friendship groups.
2. It is hypothesized that structured community friendship groups will show greater effectiveness for social capital-based outcomes compared to unstructured friendship groups.
3. Results permitting, it is hypothesized that structured community-based friendship groups will show significantly greater long-term follow up effects for social capital-based and mental health outcomes.

## Method

### Research design

Data from previous studies that have investigated mental health outcomes in friendship groups run in the community will be synthesized as part of this meta-analysis. The initial study protocol was registered with the International Prospective Register of Systematic Reviews (PROSPERO; registration number - CRD42021260639). The Preferred Reporting Items for Systematic Reviews and Meta-Analyses (PRISMA) guidelines (see Supplementary material) was used to maintain transparency and consistency in reporting standards of the proposed meta-analysis.

### Eligibility criteria

#### Inclusion criteria

No limits were placed on the language, sample size, sampling method, duration of structured or unstructured groups, and publication status of studies. The following criteria was used to guide data synthesis from studies for the meta-analysis:

- The minimum age of participants as 18 years old.
- Quantitatively designed studies (to account for previous limitations posed by systematic reviews).
- Studies that utilized either a structured or unstructured social or friendship group.
- Measured common mental health outcomes such as depression and anxiety using an evidence-based and psychometrically sound measure.
- Measured outcomes of perceived social support, loneliness, belongingness, or connection.
- Studies including sufficient information for the effect size to be calculated.
- Studies published between 1980 and June 2021.
- Original research studies.

### Procedure

#### Search strategy and mechanisms

Various online search engines were utilized for the study search. Studies extracted from each search engine were exported into the EndNote X9 software program. Search engines for this meta-analysis protocol have been guided by Flores et al. (2018) as well as those available through the Curtin University Library and were used alongside independent searches conducted through other relevant databases. The following databases were searched: PsychINFO (Ovid), MEDLINE (Proquest), AMED (Ovid), PubMed, Cochrane, ERIC (Proquest), PsycARTICLES (Ovid), PROSPERO, and Emcare (Ovid). A grey literature search was initially conducted using MedNar, Open Grey, Trove, and ProQuest dissertations and theses. However, the agreed upon search terms used across MEDLINE, PsycARTICLES, and gray literature databases returned over 20,000 results. This was determined as significantly above a targeted and reasonable number of results within the timeframe available for this meta-analysis. As such, the databases were excluded. The author lastly conducted a reverse search by hand of the reference lists of relevant studies deemed eligible for inclusion, as well as of relevant systematic reviews and meta-analyses (e.g., De Silva et al., 2005; Flores et al., 2018).

#### Search terms

A systematic review search strategy was developed by the author in collaboration with a Curtin University Research Librarian, with expertise in tailoring search strategies to each specific database. Key search words included “friendship group,” “social group,” “social capital,” “peer group,” “support group,” “social connectedness,” and “mental health.” Adapted search strategies with permitted truncations (e.g., friend* group), wildcards (e.g., connect*) and Boolean operations (e.g., friendship AND group) were used across each database. An example of the search strategy tailored to Ovid databases is presented in the Supplementary material.

#### Study selection

**Figure 1:**
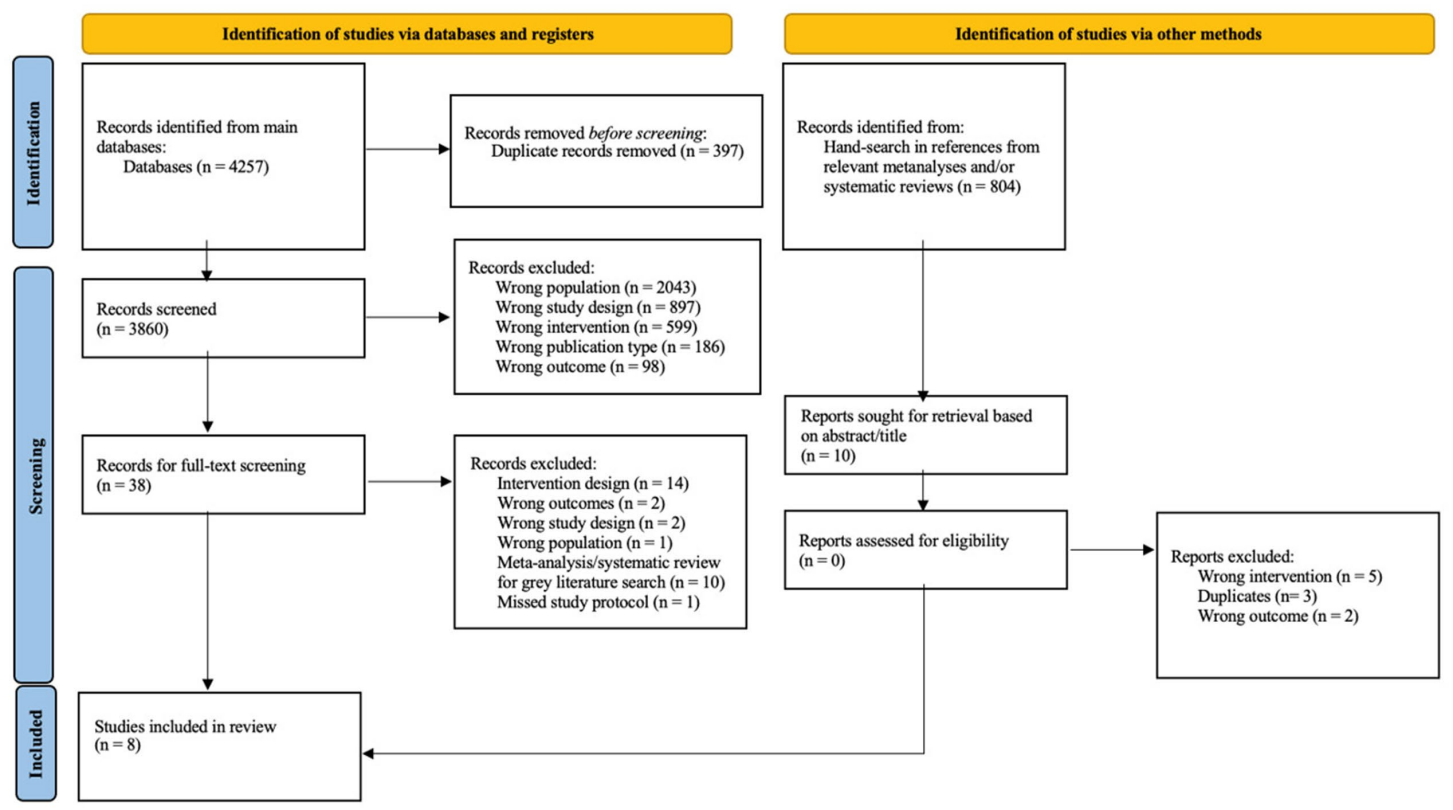
PRISMA flow of studies diagram to guide the study screening process. From Page et al. (2021).

From each database searched, 4,257 references were imported into Rayyan where study titles and abstracts were screened for initial eligibility using the predetermined inclusion criteria (see Figure 1). Three hundred and ninety seven duplicates were removed before the screening process. Next, initially eligible studies were examined and 3,823 studies were excluded after the initial abstract and title screening and were recorded in Microsoft Excel. Initial protocol for the study indicated a 30% double-screening of studies however due to time constraints and following consultation with supervisors and co-authors, a 10% double-screening was determined and conducted via an independent researcher using AS Review (Van de Schoot et al., 2020). Thirty eight studies underwent full text screening and 30 studies were excluded, leaving 8 studies. This was due to either having the wrong intervention, population, or study design, being a meta-analyses or systematic review, having a different social outcome, or the study protocol was missed. There were no studies reported from any other method, such as hand search because identified studies had the wrong intervention, were duplicates, or had the wrong outcome.

The excluded studies indicated that social support through participating in community based group interventions can improve a number of mental health and general health factors including, overcoming social isolation, acquiring health knowledge, increasing hope, improving skills to develop social relationships, and reduce of anxiety and depression (Stewart et al., 2001; Field et al., 2013; Henteleff and Wall, 2018; Lindsay-Smith et al., 2018). Although these studies do not meet the inclusion criteria they suggest the importance and benefits of community friendship groups. The issue the program targeted within the population group was also found to decrease. For example Aschbrenner et al. (2016) found obesity decreased in individuals with a diagnosed mental illness through their lifestyle intervention program, which included weight management sessions, exercise sessions, and mobile technology to increase motivation and support.

### Data screening

As per the PRISMA guidelines (Moher et al., 2009) the study inclusion process were recorded in a PRISMA ‘Flow of Studies’ diagram (see Figure 1).

### Quality evaluation criteria

The eight studies that met inclusion criteria for the proposed meta-analysis were evaluated for methodological quality using the Effective Public Health Practice Project (EPHPP) quality assessment tool (Thomas et al., 1999). The tool has been established as a tool for study quality assessment, particularly within the public health literature (Armijo-Olivo et al., 2012). The tool consists of six components (selection bias, study design, confounders, blinding, data collection method, and withdrawals and dropouts) which hold between two and four questions within each component. The EPHPP website provides a “dictionary” to guide quality evaluation which was utilized with each included study.^1^

Each component was rated as being either strong (1), moderate (2), or weak (3) in strength based on question answers. A global rating for each study quality was then compiled following structured instructions from the assessment template and dictionary guide. A global rating of “strong” represented greater study quality as assessed by the tool whilst a “weak” or “medium” reflected poorer study quality. To account for a possible low number of studies included in the final review, no studies were removed based on their global ratings of quality, however overall study quality will be used to inform the review of current research. Due to time constrictions, only the main author conducted the quality assessment of included studies. Table 1 provides the summarized quality assessment results whilst a complete table is available in the Supplementary material.

**Table 1:** Summarized quality assessment results for included studies utilizing the EPHPP.

Study	Overall rating	Selection bias	Study design	Confounders	Blinding	Data collection method	Withdrawals and dropouts
Brown et al. (2020)	Weak	Moderate	Moderate	Weak	Weak	Strong	Strong
Carandang et al. (2020)	Medium	Moderate	Strong	Strong	Weak	Strong	Strong
Harley et al. (2020)	Medium	Moderate	Moderate	Strong	Moderate	Strong	Weak
Saito et al. (2012)	Weak	Weak	Strong	Weak	Moderate	Strong	Moderate
Gleibs et al. (2011)	Strong	Moderate	Moderate	Moderate	Moderate	Moderate	Strong
Lipman and Boyle (2005)	Medium	Strong	Strong	Strong	Moderate	Strong	Weak
Cruwys et al. (2014)	Weak	Weak	Moderate	Weak	Moderate	Strong	Weak
Haslam et al. (2019)	Strong	Strong	Strong	Strong	Moderate	Strong	Moderate

Study	% Participants receiving allocated intervention or exposure?	Consistency of intervention measured?	Co-intervention or contamination likelihood?	Unit of allocation	Unit of analysis	Statistical methods appropriate?	Analysis performed by intervention allocation status rather than actual intervention?
Brown et al. (2020)	60–79%	Cannot Tell	Yes	Community	Community	Yes	No
Carandang et al. (2020)	less than 60%	Cannot Tell	Cannot Tell	Community	Community	Yes	Yes
Harley et al. (2020)	Cannot Tell	Cannot Tell	Cannot Tell	Community	Community	Yes	Yes
Saito et al. (2012)	60–79%	Cannot Tell	Yes	Community	Community	Yes	Yes
Gleibs et al. (2011)	80–100%	Cannot Tell	Cannot Tell	Organization/Institution	Organization/Institution	Yes	Yes
Lipman and Boyle (2005)	80–100%	Cannot Tell	Cannot Tell	Community	Community	Yes	No
Cruwys et al. (2014)	80–100%	Cannot Tell	Yes	Community	Community	Yes	No
Haslam et al. (2019)	80–100%	Cannot Tell	Cannot Tell	Community	Community	Yes	Yes

### Summary of quality assessment

Studies showed variety in their overall quality rating. All studies rated well for the quality of data collection tools used, whilst no included study indicated any evaluation of whether the intervention was consistently implemented over time. Three studies utilized randomization however none blinded the experimenter. Four studies utilized a control group. The remaining four studies were of a pre-post group nature, of which one utilized a gendered comparison group.

## Results

### Study characteristics

Descriptive statistics are available in Table 2 and extracted data is available in Table 3. All groups apart from Haslam et al. (2019) appeared to be “open rolling groups” with selected participants able to join in groups as they pleased rather than being required to attend. Post-treatment analyses were based on participants that attended a certain minimum number of groups, which varied in each individual study.

**Table 2:** Descriptive data from included studies following systematic search.

Study name	Country	Publication status of the study	Number of participants pre- and post-attrition	Participants mean age	Participant gender average
Brown et al. (2020)	Britain	Published	Pre-Intervention: 61. Post Intervention: 58.	34 years (SD = 6.1)	100% female
Carandang et al. (2020)	Philippines	Published	Pre intervention: 138 (68 social engagement group, 70 control). Post Intervention: 134 (66 social engagement, 68 control).	68.55 years (SD = 6.1)	70.8% female
Harley et al. (2020)	America	Published	Pre intervention: 46 participants completed main outcomes, of those, 34 completed social outcomes. Post intervention: 23 completed main outcomes, of those, 15 completed social outcomes.	Not reported	100% male
Saito et al. (2012)	Japan	Published	Pre-Intervention: 21 in intervention group and 42 in control group. Post Intervention and full follow up periods: 20 in intervention group and 37 in control group.	Intervention; 72.6 years (SD = 4.4), Control; 72.8 years (SD = 4.8)	60% female (intervention), 70% female (control)
Haslam et al. (2019)	Australia	Published	Pre-Intervention: 66 (intervention), 54 (control). Post Intervention: 46 (intervention), 53 (control).	31.06 (SD = 12.8)	64% female
Lipman and Boyle (2005)	Canada	Published	Pre-Intervention: 59 (intervention), 57 (control) Post Intervention: Intervention - 53 (after intervention), 51 (3-month follow up), 60 (6-month follow up). Control - 48 (after intervention), 41 (3-month follow up), 33 (6-month follow up).	Intervention: 32.4 years (SD = 6.7) Control: 32.3 years (SD = 6.1)	100% female
Cruwys et al. (2014)	Australia	Published	Pre-Intervention: 89. Post Intervention: 52.	44.65 (SD = 13.79)	75% female
Gleibs et al. (2011)	Britain	Published	Pre-Intervention: 30 residents from 6 different care homes. Post Intervention: 26 residents from 6 different care homes.	85.34 (SD = 7.94)	42% female

**Table 3:** Data extraction from included studies following systematic search.

Study name	Type of friendship group and intervention	Population
Brown et al. (2020)	Structured – Social Support group for mothers named “Mumspace” run by trained parent volunteers (see Bolton et al., 2020). Half group time was socializing, and half was parent-led discussion of a particular topic (e.g., motherhood, immunizations). Topics were decided by participants in quarterly meetings.	Mothers in primarily low-socioeconomic community setting of South London.
Carandang et al. (2020)	Structured – Social engagement group comprising of social events held at a community center. Social events were divided into 1.5-h batches each with 30–35 participants allocated to the group. Groups began with a prayer, then 15–20 min dancing, educational talk, group discussion and activity, interactive games, and karaoke. Health talks were run by health providers.	Community dwelling Seniors who scored above 5 on the Geriatric Depression Scale.
Harley et al. (2020)	Structured – Peer support group meetings for Black neighborhood men were facilitated by an experienced group facilitator who was a black male, began with a meal and fellowship and used a toolkit developed in Watkins et al. (2017) to guide group topics (e.g., lived experience of black men).	Black men in a 110-block area of an urban neighborhood.
Saito et al. (2012)	Structured – Group based support program of four sessions aiming to prevent social isolation and depression. First session participants socialized with other participants. Second session participants discussed the effect of relocation on their lives. Third session was finding out what information participants wanted and connection to gatekeepers. Fourth session was a community sightseeing tour of a particular city within Tokyo.	Elderly community dwellers who had recently relocated to Tokyo.
Haslam et al. (2019)	Structured: Participants engaged in a structured, 5-module, manualized group program focusing on skills training to build social group identification. Group membership noted by the authors as being part of the intervention as much as the manualized content. Group facilitated by Provisionally registered psychologists under supervision.	Individuals experiencing social isolation and either had a mental health diagnosis or met criteria for clinical depression on the Patient Health Questionnaire-9. Individuals were recruited from community and university health services.
Lipman and Boyle (2005)	Structured: Participants engaged in a social support group that also provided education via a manual regarding child-related topics (e.g., development), and maternal topics (e.g., isolation, stress). Groups were facilitated by trained “leaders” who utilized cognitive-behavioral therapy techniques and provided structured group counseling.	Single mothers with young children recruited from various community locations.
Cruwys et al. (2014)	Unstructured: Participants engaged in any of four social groups (indoor soccer, sewing, yoga, or art) run by an organization named Reclink.	Members of the community recruited through social recreation groups run by a community organization named “Reclink.”
Gleibs et al. (2011)	Unstructured: Participants engaged in gender-based groups consisting of various activities, each chosen by group members for each specific session. Participation in group sessions was voluntary and did not follow any specific program, aiming to increase social contact between participants and general wellbeing.	Care home residents from 6 different care homes.

Study name	Group timing	Methodology	Relevant outcomes assessed
Brown et al. (2020)	2 h per week over 30 months.	Cohort study	Social capital/support, maternal mental health.
Carandang et al. (2020)	3 h per week over 3 months.	Community RCT but not blinded	Geriatric depression, psychological resilience, perceived social support, loneliness.
Harley et al. (2020)	Meetings were 2 h each and met 1–2 times per month, for 13–14 months (15–16 official meetings per group). Total project for 3 years with a new group approximately each year.	Cohort study	Social support, social capital, social networks, perceived stress, self-esteem, physical and mental health.
Saito et al. (2012)	Four, 2-h sessions conducted every fortnight for 8 weeks.	Community RCT	Subjective well-being, depression, loneliness, social support measure not well-established.
Haslam et al. (2019)	Each module lasted between 60 and 90 min. The first four modules ran weekly for 4 weeks, and the fifth module was conducted 1 month after the fourth to allow for participant skills consolidation.	RCT	Depression and loneliness.
Lipman and Boyle (2005)	Weekly 1.5-h groups over 10 weeks. Participant children offered day-care whilst mother’s attended group.	RCT	Depression, self-esteem, and social support.
Cruwys et al. (2014)	Participants were deemed part of the study if they participated in the same social group at least monthly, over 3 months.	Cohort Analytic	Depression and anxiety and stress, social identification.
Gleibs et al. (2011)	Fortnightly over 12 weeks.	Cohort Analytic	Social identification, anxiety and depression.

Study name	Measures used to assess outcomes	Period of measurement (pre and post-test, and any follow up period)	Engagement
Brown et al. (2020)	Arizona Social Support Interview Schedule, Patient Health Questionnaire (PHQ-9), Generalized Anxiety Disorder Questionnaire (GAD-7).	Pre intervention and 6 months follow up after first group attendance.	72% of participants engaged with at least 5 or more sessions over the assessed 6 months.
Carandang et al. (2020)	Geriatric Depression Scale (GDS-15), Resilience Appraisal Scale (RAS-12), Duke Social Support Index (DSSI-10), UCLA Loneliness Scale (ULS-8).	Pre intervention, post intervention (3 months), and 1 month follow up.	97.1% of participants engaged within each group.
Harley et al. (2020)	Social Provisions Scale (24-Item), Perceived Stress Scale, Sampson’s Collective Efficacy Scale, Rosenburg Self-Esteem Scale, SF-12, Social Disconnectedness and Perceived Isolation Scale.	Pre intervention, Post intervention 13–14 month follow up.	Participants individually attended between 2 and 16 meetings per group. Average 7 meetings attended per participant.
Saito et al. (2012)	Life Satisfaction Index (LSI-A), Geriatric Depression Scale (GDS), Ando-Osada-Kodama (AOK) Loneliness Scale.	Pre intervention, post intervention, 1 month follow up, and 6 month follow up.	65% of intervention group participants attended all four sessions.
Haslam et al. (2019)	UCLA Loneliness Scale, Depression subscale from the Depression Anxiety and Stress Scale (DASS-21).	Pre-intervention and post-intervention (flexible follow-up period recorded between 55 and 204 days).	Intervention: 69% of baseline participants completed follow-up measures. Control: 98% of baseline control participants completed follow-up measures.
Lipman and Boyle (2005)	Center of Epidemiologic Studies Depression Scale (CES-D), Rosenberg Self-esteem Scale, Social Provisions Scale.	Pre-intervention, post-intervention, and follow up periods of 3- and 6-months post-intervention.	Intervention: 90% engaged at post-intervention data collection, 86% at 3 month follow up, and 84% at 6 month follow up. Control: 84% engaged at post-intervention data collection, 71% at 3 month follow up, and 57% at 6 month follow up.
Cruwys et al. (2014)	Depression Anxiety and Stress Scale (DASS-21), Social Identification measured using four adapted items from Doosje et al. (1995) with appropriate Cronbach’s Alpha (𝛼 = 0.91).	Pre-intervention and post-intervention.	58.4% of participants measured pre-intervention were retained at post-intervention data collection.
Gleibs et al. (2011)	Social Identification Scale (two items that were highly correlated), Hospital Anxiety and Depression Scale.	Pre intervention, mid-intervention (4 weeks), post intervention.	13% of participants unable to finish study due to ill health or unavailability to complete post-intervention measures.

Study name	Results for mental health and/or general wellbeing outcome measures	Results for social capital-based outcome measures (e.g., connectedness or social wellbeing)
Brown et al. (2020)	Statistically significant group differences found for PHQ results (*p* < 0.001, *t* = 3.78, *d* = 0.44) and for GAD-7 results (*p* ≤ 0.001, *t* = 3.36, *d =* 0.37). Participants who scored above clinically significant thresholds shows statistically significant declines in reported symptoms after intervention across PHQ (*p* < 0.001, *t =* 6.17) and GAD-7 measures (*p* ≤ 0.001, *t =* 6.57). Participants who did not score above clinical threshold did not show a statistically significant change in symptoms after intervention.	No significant difference in participant’s total network size, however statistically significant difference found for participant’s total network satisfaction (*p* = 0.04, *t* = −2.06, *df* = 57). No effect sizes provided.
Carandang et al. (2020)	Social engagement group reported statistically significant reductions in depression symptoms (*p* < 0.00,1, *d* = −1.10), compared to control group.	Social engagement group reported statistically significantly improvements in participant perceived social support (*p* < 0.00, *d* = 1.07) compared to control group.
Harley et al. (2020)	After adjustment for potential confounding factors: Statistically significant results for improvements in perceived stress (*β* = −3.41, *p* = 0.047). No significant change for self-esteem. No effect sizes provided.	Statistically significant results for increases in social support (provision) (*β* = 5.20, *p* = 0.027), particularly for reassurance of worth (*p* = 0.005), and reliable alliance (*p* = 0.03). No significant change for social network contacts or number of friends. No effect sizes provided.
Saito et al. (2012)	Statistically significant results were found for increases in subjective well-being (*p* = 0.04). No significant change for depression.	Statistically significant results were found for increases in social support (*p* = 0.013) and reductions in loneliness (*p* = 0.01). No significant change for informal social network measure.
Haslam et al. (2019)	Depression symptoms found to have significant decrease in intervention group (*d* = 0.34) as well as the control condition (*d* = 0.34). Difference between the intervention and treatment as usual group was not measured.	No social capital outcomes were measured.
Lipman and Boyle (2005)	Social group participation accelerated improvements in depressed mood compared to control (*d* = 0.41).	No significant difference for social support levels across both groups.
Cruwys et al. (2014)	Significant decline found for participant depression symptoms post-treatment to a below clinical cut-off level for mild depression (*d* = 0.47). Greater social identification with Reclink groups predicted stronger decline in depression symptoms (ƞ_p_^2^ = 0.08).	Social identification was not investigated or reported as a direct outcome from participant group involvement. Social identification as a mediator was reported (see depression outcomes).
Gleibs et al. (2011)	Statistically significant decreases found in depression (*p* = 0.03, ƞ_p_^2^ = 0.19) scores for male participants compared to female. No significant changes for depression symptoms in female participants. Reported significant decrease in anxiety symptoms for males (*p* = 0.09) but not for females.	Statistically significant increases found in social identification for male participants compared to female (*p* = 0.09, ƞ_p_^2^ = 0.14). No significant changes for social identification in female participants.

#### Structured friendship groups

Structured groups ranged between 1 and 3 h per week to per month. Participants of included studies varied in country of origin and languages. There was a different in average age, indicated by the varied target population between studies. For example Brown et al. (2020) participants were young mothers that had an average age of 34 (SD = 6.1) and Carandang et al. (2020) participants were seniors that had an average age of 68.55 (SD = 6.1). Mixed-gendered groups were utilized by half of the included studies, however it appeared that over 60% of participants were female, within each mixed-gendered study. Specific-gender groups appeared to target the structured friendship group around gender-specific topics (e.g., mother’s groups).

All groups followed either a manualized format or a schedule aligned with time to form friendships before or after structured activities and discussions. Four of the overall six studies utilized a control group as part of randomized control trials.

Groups varied in overall length but appeared to run between 1 and 3 h per group session and varied between weekly to monthly attendance. The timeline of the group ranged between 2 months and 13–14 months [Brown et al. (2020) ran for 30 months overall, however post-intervention evaluations were conducted 6 months after participants initially began the group].

##### Mental health outcomes

All studies utilized measures for depression symptoms and used population specific measures (e.g., the Geriatric Depression Scale for senior populations). Haslam et al. (2019) reported measuring social anxiety symptoms and Brown et al. (2020) also reported generalized anxiety symptoms. However, the decision was made to exclude this outcome measurement as other included studies did not directly explore social anxiety within the structured groups. Effect sizes ranged from small (Lipman and Boyle, 2005; Haslam et al., 2019; Brown et al., 2020) in the overall treatment effect compared to control, to large (Brown et al., 2020) for only participants scoring above the clinical range of depression symptoms (Carandang et al., 2020). No effect sizes were reported by Saito et al. (2012) and Harley et al. (2020) reported no significant changes for depression symptoms.

##### Social capital outcomes

All structured group studies, except for Saito et al. (2012) and Haslam et al. (2019), utilized perceived social support measures. Saito et al. (2012) and Haslam et al. (2019), in addition to Carandang et al. (2020), further measured loneliness using the UCLA Loneliness Scale (Russell, 1996) or adapted versions. A mix of results was indicated for social support. Carandang et al. (2020) reported a large effect for perceived social support. Lipman and Boyle (2005) did not find a significant effect whilst Brown et al. (2020) reported a significant effect found for participant’s network satisfaction but not for changes to participant social network size. Saito et al. (2012) and Harley et al. (2020) found significant effects for increased perceived social support but no significant changes in participant social network size.

#### Unstructured friendship groups

Two studies were included overall as containing unstructured friendship groups. Both studies were of a pre-post treatment design with no control group. Unstructured groups ranged between fortnightly to at least monthly attendance, both lasting approximately 3 months overall. Neither study mentioned the specific time length of respective group sessions. A general summary of participant ethnicities was not provided by either study. As both studies targeted different populations, age ranges varied significantly but were reflective of each target population. Members of a community recruited through social recreation groups had an average age of 44.65 (SD = 13.79), compared to care home residents who had an average age of 85.35 (SD = 7.94). Both studies used mixed-gendered groups. Cruwys et al. (2014) reported 75% of their participants were female and Gleibs et al. (2011) reported 42% of their participants were female. Groups appeared to be unstructured in the content of the group however each involved activities (e.g., sports) decided by participants from a selection provided on the day, and for the primary purpose of friendship.

##### Mental health outcomes

Both studies utilized depression and anxiety symptom screening measures (Hospital Anxiety and Depression Scale, Snaith, 2003; DASS-21, Sinclair et al., 2012), however, Cruwys et al. (2014) only reported outcomes of the depression subscale. Both groups reported differing outcome effects; Gleibs et al. (2011) reported a large effect on decreasing depression and anxiety symptoms in male participants only, whilst Cruwys et al. (2014) reported a medium effect of decreasing depression symptoms.

##### Social capital outcomes

Both studies used social identification measures. However, Cruwys et al. (2014) did not report social identification as a direct outcome and rather stated social identification as a predictor of decreased participant depression symptoms. Gleibs et al. (2011) reported a large effect for greater social identification in male participants, but not female.

### Meta-analysis

In keeping with recommendations from Hall and Rosenthal (2018), the meta-analysis was conducted using a random effects (RE) model in order to account for the variability in study effect sizes, methodological differences within each structured group intervention, and sample population differences. Given the small number of studies included, Hedge’s *g* was the effect size used due to its more conservative nature (Borenstein et al., 2021). Due to the variability in the measurements used for study outcomes of interest, standardized mean differences were used in the meta-analysis (see Faraone, 2008). Further, as depression was the primary mental health outcome reported across the included studies, we used that as the mental health outcome. Three studies indicated loneliness as an outcome measured and were included as a separate meta-analysis for social outcomes.

Given the differences in research design of the final included studies, a meta-analysis was only conducted on studies with between-group differences (i.e., differences at post-treatment between control and treatment groups). An effect size from a single pre-post group study will have a different meaning to that of an effect size between-groups (Lipsey and Wilson, 2001). Randomized study designs with a control group reduce the variance in the result that may otherwise present in single pre-post study designs (Hall and Rosenthal, 2018). As such, only the studies of Lipman and Boyle (2005), Haslam et al. (2019), and Carandang et al. (2020) were included in the meta-analysis component of the study (see Table 4). As no unstructured friendship group studies indicated a control group no meta-analysis was conducted for unstructured friendship groups. After two email attempts over several weeks by the author for additional data, Saito et al. (2012) was removed from meta-analysis inclusion as the post-treatment data required was not available in the published study. As Haslam et al. (2019) only reported outcomes for depression, the study was not included in the meta-analysis of social capital outcomes. The Cochrane’s *Q* heterogeneity test, was used to show the degree of study heterogeneity within each meta-analysis (Higgins and Thompson, 2002). As recommended by Higgins and Thompson (2002) and Higgins et al. (2003), an *I*^2^ value of >40% indicates between moderate to substantial heterogeneity. The overall results across depression, social, and loneliness outcomes are presented in Table 4.

**Table 4:** Summary statistics of the final random effects (RE) model for depression, social, and loneliness outcomes from included studies.

Outcomes	No. included studies	RE model combined effect size (SE)	*Z*-value	*Q* test for homogeneity (value of *p*)	*I^2^*	Description
Depression	2	0.32 (0.09)	3.56	0.37 (*p* = 0.54)	0.00%	Low between study variance
Social (general)	2	0.67 (0.53)	1.25	15.35 (*p* = 0.00)	93.49%	High between study variance
Loneliness	2	0.40 (0.25)	1.64	3.05 (*p* = 0.08)	67.21%	High between study variance

#### Depression

Contact was made to authors of Haslam et al. (2019) to obtain post-treatment means and standard deviations. Post-treatment data obtained was inputted alongside existing data of the other studies and into a validated, open-source, meta-analysis tool known as Meta Essentials (Suurmond et al., 2017).

**Figure 2:**
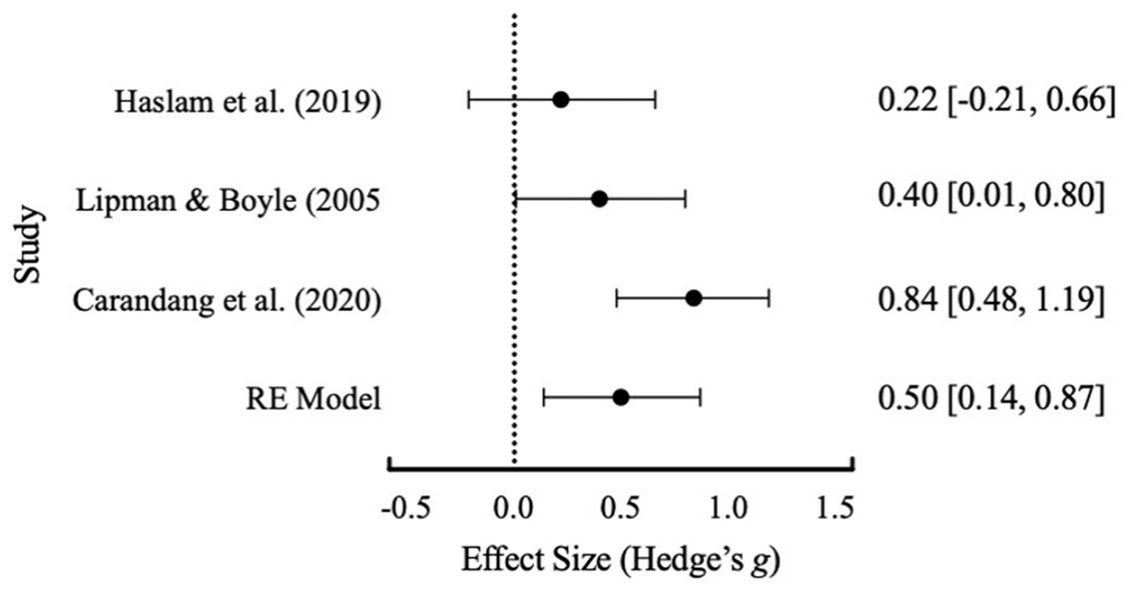
Effect size distribution of the RE model with 95% confidence intervals for depression before the exclusion of Carandang et al. (2020).

**Figure 3:**
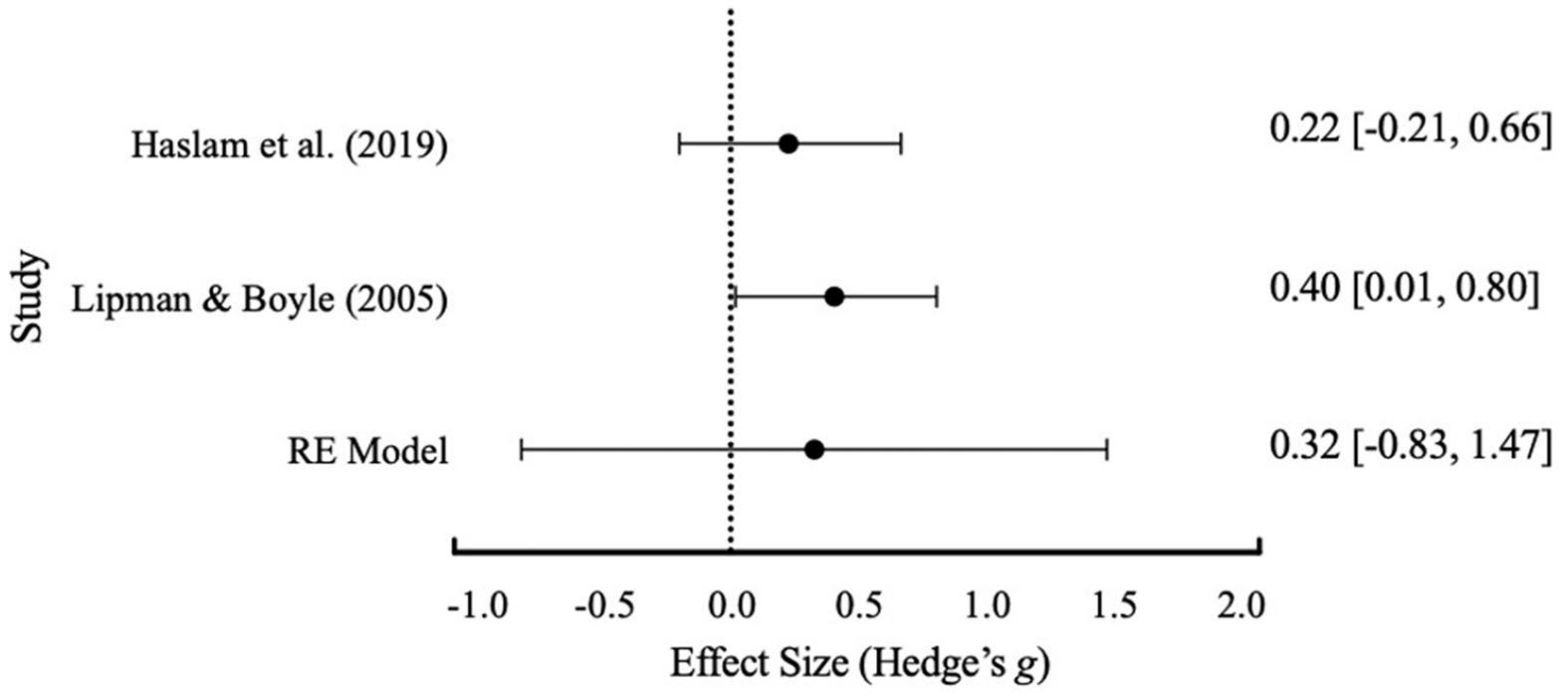
Effect size distribution of the RE model with 95% confidence intervals for depression after the exclusion of Carandang et al. (2020).

An initial meta-analysis with the three studies of Lipman and Boyle (2005), Haslam et al. (2019), and Carandang et al. (2020), revealed a Cochrane’s *Q* test (*p* = 0.074) with an *I*^2^ value of 61.14% that suggested significant heterogeneity between studies (see Table 5 for full results table and Figure 2 for forest plot). Whilst the usual methodology would then suggest further subgroup analyses are warranted there were not enough studies available to conduct subgroup analyses (Mikolajewicz and Komarova, 2019). A Failsafe N test using Rosenthal (1979) was conducted and indicated that 19 additional studies were required to reduce overall heterogeneity. Based on the data extraction, Carandang et al. (2020) was identified by the lead author as having substantially higher average participant age and was removed from the analysis. Following removal, the results (see Table 4) suggested no heterogeneity and a more accurate overall effect size. This was further visually evident within Figure 3. Based on interpretation guidelines for Hedge’s *g* (see Lakens, 2013) a small effect was indicated the overall RE model. Overall, structured friendship groups included in this meta-analysis appeared to have significantly decreased participant depression symptoms as an indicator of mental health.

**Table 5:** Summary statistics of the final random effects (RE) model for depression prior to exclusion of Carandang et al. (2020).

Outcomes	No. studies	RE Model Combined effect size* (SE)	Study effect size* (SE)	95% C.I of effect size	*Z*-score	*Q* test for homogeneity (value of *p*)	*I^2^*	Description
Depression	3	0.50 (0.18)		−0.29, 1.29	2.74	5.28 (0.07)	62.12%	High between study variance
Carandang et al. (2020)			0.84 (0.18)	0.49, 1.20				
Lipman and Boyle (2005)			0.40 (0.20)	0.01, 0.80				
Haslam et al. (2019)			0.22 (0.22)	−0.21, 0.66				

*Hedge’s *g*.

#### Social outcomes

**Figure 4:**
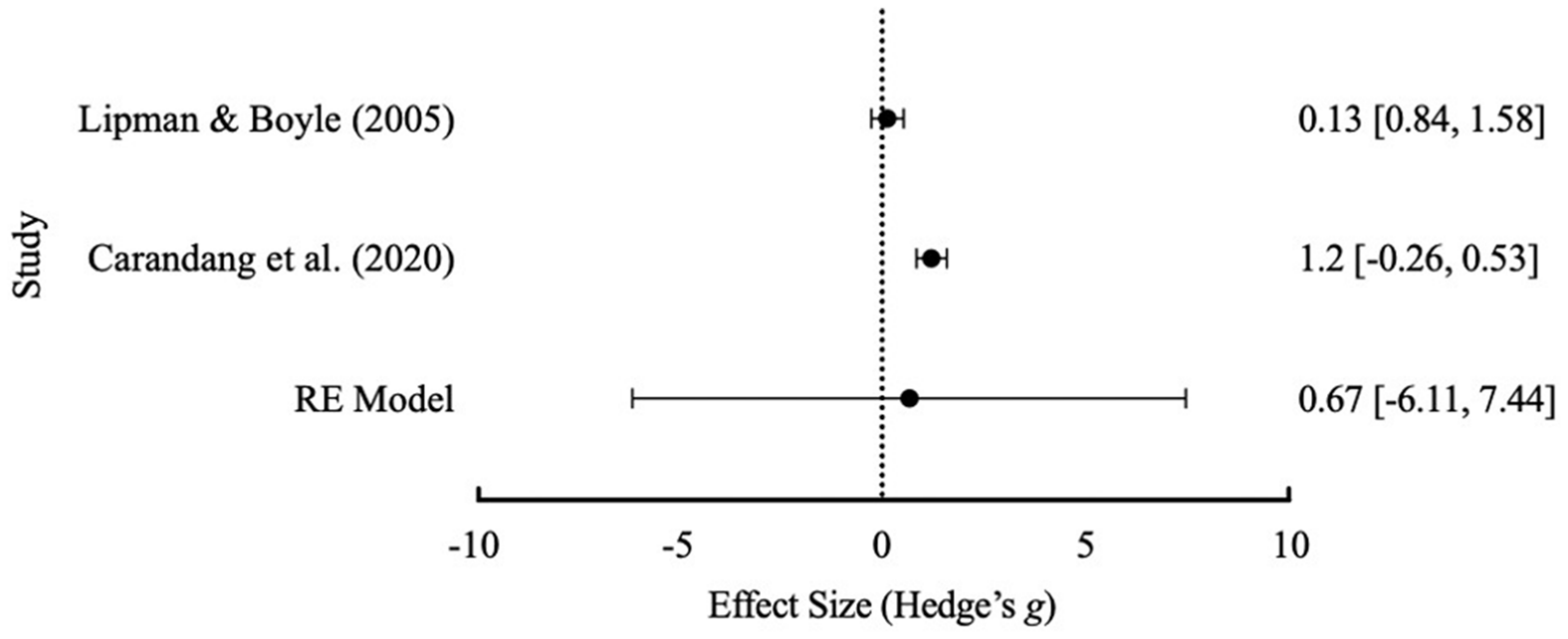
Effect size distribution of the RE model with 95% confidence intervals for general social outcomes.

**Figure 5:**
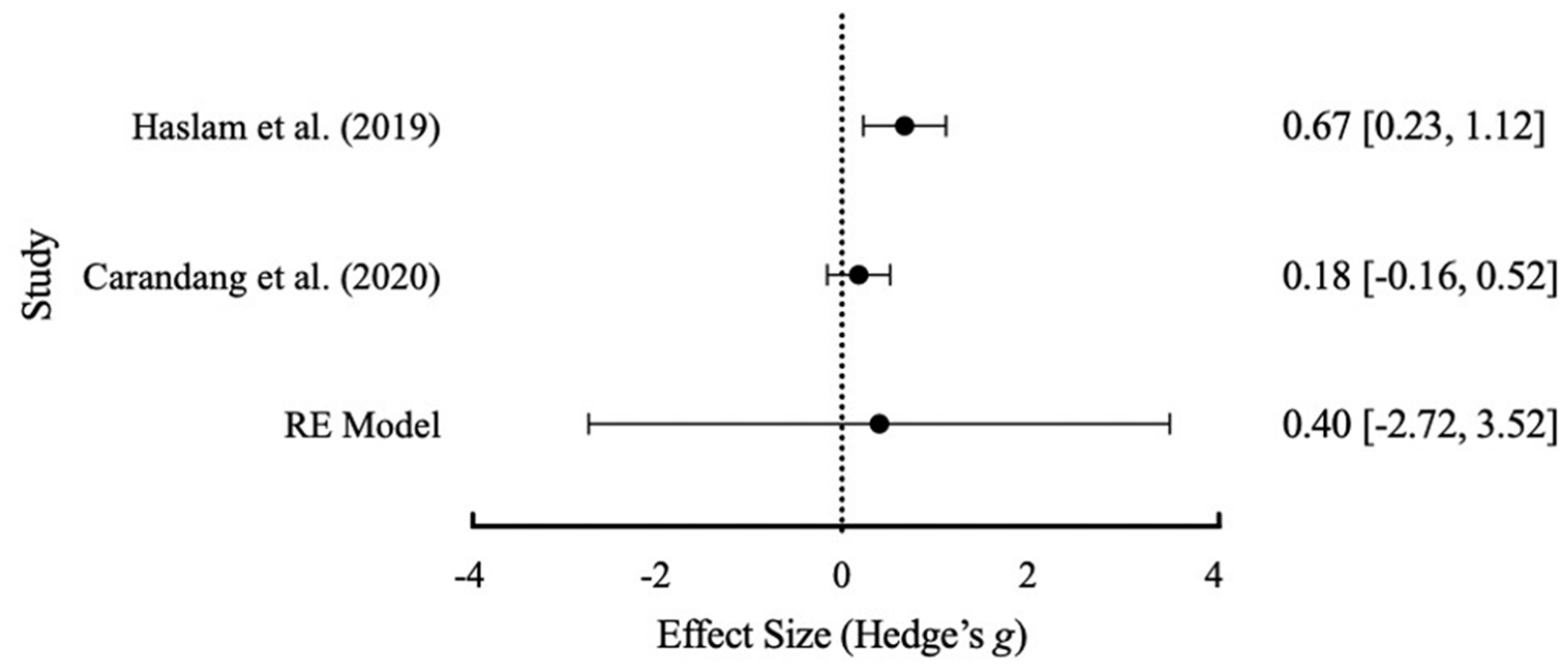
Effect size distribution of the RE model with 95% confidence intervals for loneliness.

As there was available data for loneliness-specific outcomes a brief meta-analysis was also conducted to investigate loneliness as part of social capital outcomes. Initial meta-analysis results (see Table 4) indicated there was significant heterogeneity between studies for general social outcomes and loneliness. There were not enough included studies to warrant a subgroup analysis or any single-study exclusions therefore none were conducted. A Failsafe *N* test was conducted and indicated that 17 additional studies were required to reduce overall heterogeneity for general social outcomes and 4 additional studies for loneliness. Due to the significant Cochrane’s *Q* test and no available studies to exclude, the overall effect sizes from either meta-analysis cannot be confidently interpreted without significant bias. A distribution of the effect sizes is shown in Figure 4 for general social capital outcomes and Figure 5 for loneliness as a social capital-based outcome.

#### Follow-up outcomes of social capital and mental health

Within the studies utilized a follow-up period, there was not enough concurrent follow-up data available to conclude the third hypothesis. Lipman and Boyle (2005), Saito et al. (2012), and Carandang et al. (2020) included follow-up periods post-treatment. However, Carandang et al. (2020) included a 1-month post-treatment follow up period whilst Lipman and Boyle (2005) reported two follow up periods of 3- and 6-months post-treatment. As discussed, post-treatment data was not available from Saito et al. (2012). The decision was therefore made to not conduct a follow-up data meta-analysis due to the vast variability in the time periods post-treatment.

## Discussion

The present paper aimed to address previous knowledge gaps of structured and unstructured community friendship groups and efficacy on mental health and social outcomes in community participants. A systematic search was conducted through various databases followed by a meta-analysis of the results to determine collective outcomes. Overall, eight studies were included in the review; two were classified as unstructured friendship group studies and six as structured friendship group studies. There was not enough available data to conduct a comparison between structured and unstructured groups and their effects on mental health or social capital outcomes. Therefore, this significantly limited our ability to determine our hypotheses. However, as the original aim of this paper was to also add to available literature on friendship groups and outcomes, meta-analyses were conducted where appropriate for each hypothesis. Further interpretations of the results of the meta-analysis are limited due to available data and overall number of included studies.

### Hypothesis 1: structured community friendship groups will show greater effectiveness for mental health outcomes compared to unstructured friendship groups.

There was not enough available data to conduct a comparison between structured and unstructured groups and their effects on mental health outcomes. Given the limited literature summarizing structured friendship group outcomes, a meta-analysis of mental health outcomes (depression) from structured friendship groups was conducted. The meta-analysis contained three of the four included structured friendship group studies that included a control-group. This was due to insufficient post-treatment data made available to the author from the lead author of Saito et al. (2012). Following final exclusion of Carandang et al. (2020), meta-analysis results supported a small effect of structured friendship groups in reducing participant reported depression. The small effect found shows promising support for structured friendship groups as an intervention option. However, the limited number of studies and limited data available for unstructured friendship groups for comparison restrict the generalizability of our results (Mikolajewicz and Komarova, 2019).

Despite the limited generalizability, the initial small effect found shows promise for structured friendship groups as a possible community-based intervention for depression symptoms in participants. Initial results also appear to support social identification theory (Tajfel and Turner, 1979; Tajfel and Turner, 1986) in that social identification with individuals in a group may provide the opportunity for social capital and support through friendships (see Postmes et al., 2019). Friendship groups may contribute to a reduction in depression symptoms through a “social cure” promotion of adjustment and coping (Jetten et al., 2011). In doing so, friendship groups may also offer a “behavioral activation” effect often utilized in Cognitive Behavioral Therapy that promotes positive emotions and self-mastery to counteract common depressive symptoms (American Psychiatric Association, 2013). Structured groups specifically designed to teach social skills to enhance social capital may be essential to enhancing the “behavioral activation” component further (e.g., Haslam et al., 2016, 2019). Social skills training may further reduce maladaptive cognitions associated with perceived social disconnectedness. These cognitions (e.g., “they hate me”) can often lead to individuals perceiving loneliness and social disconnectedness despite identifying several social connections (Masi et al., 2011).

Initial support for structured groups affecting depression symptoms also indicates promise for more community groups to utilize friendship groups as a lower cost intervention. The promising results in this meta-analysis also add to post-COVID19 literature for promising interventions for depression after social-isolation posed by COVID-19 restrictions (Williams et al., 2021). Future research may also consider investigating the effects of structured online friendship groups in comparison to in-person groups due to greater accessibility to individuals with disabilities or residing in regional areas.

Initial results whilst investigating the first hypothesis indicate support for additional research to be conducted with the community friendship groups. There remains considerable research space for the effectiveness of structured friendship groups to be investigated through clinical or randomized control trials to determine more meaningful effects compared to single pre-post trials. This includes conducting follow-up periods to add knowledge to whether effects are maintained.

### Hypothesis 2: structured community friendship groups will show greater effectiveness for social capital outcomes compared to unstructured friendship groups

There was also not enough available data to conduct a comparison between structured and unstructured groups and their effects on social capital outcomes. As before, meta-analyses were attempted with available data to address literature limitations. The second study hypothesis was investigated with two studies (Lipman and Boyle, 2005; Carandang et al., 2020). However, results could not be generalizable across either general social capital or loneliness outcomes due to significant heterogeneity (*I^2^* value of 61.14%) found between studies upon initial analysis. Further subgroup analyses were not conducted due to the small number of studies. Independently, Carandang et al. (2020) reported their respective structured friendship groups had a significant large effect size for general social outcomes but a less than small effect for loneliness. Lipman and Boyle (2005) reported a small effect size, on participant perceived social support, and Haslam et al. (2019) reported a medium effect for loneliness reduction in participants. As Carandang et al. (2020) was excluded in the analyses for the first hypothesis due to a substantially different average participant age, heterogeneity may be due to widely different participant ages and samples (e.g., seniors vs. mothers). Both analyses were further limited with the exclusion of Saito et al. (2012) as a further sample due to non-available post-treatment data. Due to the limitations present it is difficult to generalize these results to current literature. With available individual data of the included studies (see Table 2), it appears that social capital outcomes within friendship groups remain inconsistent. Limitations with regards to the first and second hypotheses are discussed below.

### Hypothesis 3: long-term follow-up effects of friendship groups

As discussed, a limited number of studies were available for meta-analysis. Within those that were available, there was not enough concurrent follow-up data available to conclude the third hypothesis. Implications and limitations are discussed below.

### Implications

Included studies indicated that since Flores et al. (2018), literature has expanded in investigating social capital and mental health outcomes from community-based groups. To our knowledge this is the first review to investigate community-based friendship groups specifically. Ultimately, this review has indicated the need for further research to investigate the mental health and social capital outcomes of structured and unstructured friendship groups. In addition to the research implications discussed for each hypothesis above, practical implications are discussed below.

Included studies in this review also showed a mix of gender specific groups and mixed gendered groups. Mixed groups were more common amongst senior age population-based studies, which reflects research supporting loneliness as a primary concern amongst the elderly population (Poscia et al., 2018). Populations in non-senior population-based studies utilized participants from low-socioeconomic neighborhoods (e.g., Brown et al., 2020; Harley et al., 2020), or community-based medical or social services (e.g., Lipman and Boyle, 2005). This appeared to reflect the vulnerability of low-socioeconomic neighborhoods to isolation and mental health issues (Hill and Maimon, 2013; Tabatabaei-Jafari et al., 2021) and the likely need for friendship groups within these environments. Importantly, friendship groups (unstructured or structured) within the community appear to have practical implications of creating purpose-built environments for individuals to gain social capital knowing other individuals are motivated by the same purpose. Gendered groups within low-socioeconomic neighborhoods appeared to have social capital improvements, likely due to same-gender issues being present. This appeared to allow for greater participant bonding and structured information dissemination (e.g., psychoeducation for mothers, Lipman and Boyle, 2005; Brown et al., 2020). Ongoing research and application of gendered (or gender-diverse), faith-based, or specific interest groups may create accessibility to greater social networks for commonly isolated populations such as refugees, culturally diverse individuals, and LGBTIQA+ individuals. Important factors to consider within frequency of evaluated friendship groups is participant recruitment and participant willingness to increase social capital (Villalonga-Olives and Kawachi, 2017).

### Limitations

General limitations to this study include limited time impacting the decision to exclude several databases that produced a generous number of results. The majority of the excluded databases were grey literature and student dissertations that may have addressed possible other included studies that were not peer-review published.

Importantly, a large limitation to this paper was the limited number of available studies focusing on community-based friendship groups. Several reasons are discussed that may have contributed to this limited availability. Studies included in this paper indicated structured community-based friendship groups were more likely to be structured in nature than unstructured. Reasons for this difference may include that unstructured friendship groups run by community organizations may not be evaluated due to the unstructured and informal nature of the groups, or their evaluation may not be published and kept internal if conducted. For example, BeFriend project^2^ is located in Perth, Australia, and includes several unstructured community friendship groups (e.g., picnics) across various metropolitan suburbs in Perth. To the author’s knowledge, no reports from the BeFriend project are published or publicly available that evaluate quantitative outcomes of these unstructured friendship groups. Of the included structured friendship groups in this study, all notably utilized structured psychoeducation and skills training with measurable outcomes related to group content (e.g., Haslam et al., 2019). Comparatively, the informal nature of the unstructured friendship group may present unknown participant content or relationships that are unable to be captured as a cause-and-effect on participant social capital and mental health outcomes. For example, two unstructured friendship group participants with greater confidence in their social skills may report stronger social capital and mental health outcomes than other participants who do not. Further issues of evaluation within unstructured community groups may include limited research priority and the time availability of individuals involved to organize required measures and their administration (Sanson-Fisher et al., 2007). Increasing awareness of these possible factors may encourage research collaboration pathways to investigate unstructured community groups effectiveness.

Further, during the search we noted a number of studies that reflected qualitative outcome measures of structured and unstructured groups that were excluded before finalization due to the quantitative nature of this study protocol (e.g., Logie et al., 2016). This may suggest that greater evaluation of the proposed hypotheses should be considered to produce more robust results within unstructured and structured friendship group research. Future research should consider investigating a review of qualitative outcomes proposed in friendship groups to add to the body of literature available for community-based interventions.

As expected, the definition of “friendship groups” varied widely amongst searched papers. Studies excluded from the present review included those that described structured “friendship” groups as upskilling participants in making friends/forging stronger connections within their existing social networks rather than providing a space for participants to make friends *within* the group (Martina and Stevens, 2006). Although friendship may occur within the group as a by-product, often this is evaluated as a secondary variable and may be confounded by the focus on the existing social network of the participant (Boda et al., 2020). Further, given that grey literature was excluded from this meta-analysis due to a significant number of results, there may be scope for researchers to investigate results for further friendship group studies.

## Conclusion

Overall, due to limited number of structured and unstructured friendship group data available, none of the proposed hypotheses were able to be determined in this meta-analysis. Initial results did indicate support for structured friendship groups having a small effect for reducing depression symptoms. Results for social capital-based outcomes were unable to be generalized due to significant heterogeneity between studies. The meta-analysis for depression (as the mental health determinant) outcomes was underpowered with two studies. However, initial results for the effect of structured friendship groups on depression do show support for social identification theory and the influence of social capital on individual wellbeing and mental health. Results also indicate that structured community friendship groups may provide a cost-effective and accessible space for individuals to access a preventative or preliminary intervention for mental health. Consequently this review has made a significant contribution beyond previous reviews, as it has provided evidence that structured community friendship groups are a potential intervention to improve mental health in the community and further investigation into this is required. Ultimately, there remains an important research gap in the effects of community-based structured and unstructured friendship groups on mental health and social capital outcomes.

## Data availability statement

The original contributions presented in the study are included in the article/Supplementary material, further inquiries can be directed to the corresponding author.

## Author contributions

MG conceptualized the research and was the principal author of the manuscript. RR, LM, and VM supervised the project and provided feedback at all stages. LM and VM acted as statistical consultants for the meta-analysis. RM provided feedback and amended the manuscript for publication.

## Footnotes

^1^see https://www.ephpp.ca/quality-assessment-tool-for-quantitative-studies/

^2^https://befriend.org.au
