# Supplementary material for: The effectiveness of community friendship groups on participant social and mental health: a meta-analysis
Source: Front Psychol. 2023 Dec 7;14:1078268. doi: 10.3389/fpsyg.2023.1078268 (PMC10733530; doi:10.3389/fpsyg.2023.1078268)
Supplement: Supplementary file 1 [file Table_1.DOCX]

# Supplementary Material

PRISMA Guidelines statement

| **Section and Topic** | **Item #** | **Checklist item** | **Location where item is reported** |
| --- | --- | --- | --- |
| **TITLE** | | | (Page no.) |
| Title | 1 | Identify the report as a systematic review/meta-analysis | 1 |
| **ABSTRACT** | | |  |
| Abstract | 2 | See the PRISMA 2020 for Abstracts checklist. | 2 |
| **INTRODUCTION** | | |  |
| Rationale | 3 | Describe the rationale for the review in the context of existing knowledge. | 6-7 |
| Objectives | 4 | Provide an explicit statement of the objective(s) or question(s) the review addresses. | 7 |
| **METHODS** | | |  |
| Eligibility criteria | 5 | Specify the inclusion and exclusion criteria for the review and how studies were grouped for the syntheses. | 8 |
| Information sources | 6 | Specify all databases, registers, websites, organisations, reference lists and other sources searched or consulted to identify studies. Specify the date when each source was last searched or consulted. | 8-9 |
| Search strategy | 7 | Present the full search strategies for all databases, registers and websites, including any filters and limits used. | 9 |
| Selection process | 8 | Specify the methods used to decide whether a study met the inclusion criteria of the review, including how many reviewers screened each record and each report retrieved, whether they worked independently, and if applicable, details of automation tools used in the process. | 9-10 |
| Data collection process | 9 | Specify the methods used to collect data from reports, including how many reviewers collected data from each report, whether they worked independently, any processes for obtaining or confirming data from study investigators, and if applicable, details of automation tools used in the process. | 10 |
| Data items | 10a | List and define all outcomes for which data were sought. Specify whether all results that were compatible with each outcome domain in each study were sought (e.g. for all measures, time points, analyses), and if not, the methods used to decide which results to collect. | 8 |
|  | 10b | List and define all other variables for which data were sought (e.g. participant and intervention characteristics, funding sources). Describe any assumptions made about any missing or unclear information. | 12 |
| Study risk of bias assessment | 11 | Specify the methods used to assess risk of bias in the included studies, including details of the tool(s) used, how many reviewers assessed each study and whether they worked independently, and if applicable, details of automation tools used in the process. | 10-11 |
| Effect measures | 12 | Specify for each outcome the effect measure(s) (e.g. risk ratio, mean difference) used in the synthesis or presentation of results. | 11-12 |
| Synthesis methods | 13a | Describe the processes used to decide which studies were eligible for each synthesis (e.g. tabulating the study intervention characteristics and comparing against the planned groups for each synthesis (item #5)). | 11-12 |
|  | 13b | Describe any methods required to prepare the data for presentation or synthesis, such as handling of missing summary statistics, or data conversions. | 11-12 |
|  | 13c | Describe any methods used to tabulate or visually display results of individual studies and syntheses. | 11-12 |
|  | 13d | Describe any methods used to synthesize results and provide a rationale for the choice(s). If meta-analysis was performed, describe the model(s), method(s) to identify the presence and extent of statistical heterogeneity, and software package(s) used. | 15-17 |
|  | 13e | Describe any methods used to explore possible causes of heterogeneity among study results (e.g. subgroup analysis, meta-regression). | n/a |
|  | 13f | Describe any sensitivity analyses conducted to assess robustness of the synthesized results. | 15-17 |
| Reporting bias assessment | 14 | Describe any methods used to assess risk of bias due to missing results in a synthesis (arising from reporting biases). | n/a |
| Certainty assessment | 15 | Describe any methods used to assess certainty (or confidence) in the body of evidence for an outcome. | 16-17 |
| **RESULTS** | | |  |
| Study selection | 16a | Describe the results of the search and selection process, from the number of records identified in the search to the number of studies included in the review, ideally using a flow diagram. | 12 |
|  | 16b | Cite studies that might appear to meet the inclusion criteria, but which were excluded, and explain why they were excluded. | 12 |
| Study characteristics | 17 | Cite each included study and present its characteristics. | 12-14 |
| Risk of bias in studies | 18 | Present assessments of risk of bias for each included study. | 12-14 |
| Results of individual studies | 19 | For all outcomes, present, for each study: (a) summary statistics for each group (where appropriate) and (b) an effect estimate and its precision (e.g. confidence/credible interval), ideally using structured tables or plots. | 16-17 |
| Results of syntheses | 20a | For each synthesis, briefly summarise the characteristics and risk of bias among contributing studies. | 16-17 |
|  | 20b | Present results of all statistical syntheses conducted. If meta-analysis was done, present for each the summary estimate and its precision (e.g. confidence/credible interval) and measures of statistical heterogeneity. If comparing groups, describe the direction of the effect. | 16-17 |
|  | 20c | Present results of all investigations of possible causes of heterogeneity among study results. | 16-17 |
|  | 20d | Present results of all sensitivity analyses conducted to assess the robustness of the synthesized results. | 16-17 |
| Reporting biases | 21 | Present assessments of risk of bias due to missing results (arising from reporting biases) for each synthesis assessed. | 16-17 |
| Certainty of evidence | 22 | Present assessments of certainty (or confidence) in the body of evidence for each outcome assessed. | 16-17 |
| **DISCUSSION** | | |  |
| Discussion | 23a | Provide a general interpretation of the results in the context of other evidence. | 17-22 |
|  | 23b | Discuss any limitations of the evidence included in the review. | 22-24 |
|  | 23c | Discuss any limitations of the review processes used. | 22 |
|  | 23d | Discuss implications of the results for practice, policy, and future research. | 21-22 |
| **OTHER INFORMATION** | | |  |
| Registration and protocol | 24a | Provide registration information for the review, including register name and registration number, or state that the review was not registered. | 8 |
|  | 24b | Indicate where the review protocol can be accessed, or state that a protocol was not prepared. | 7-8 |
|  | 24c | Describe and explain any amendments to information provided at registration or in the protocol. | n/a |
| Support | 25 | Describe sources of financial or non-financial support for the review, and the role of the funders or sponsors in the review. | n/a |
| Competing interests | 26 | Declare any competing interests of review authors. | n/a |
| Availability of data, code and other materials | 27 | Report which of the following are publicly available and where they can be found: template data collection forms; data extracted from included studies; data used for all analyses; analytic code; any other materials used in the review. | Appendices and supplementary material |


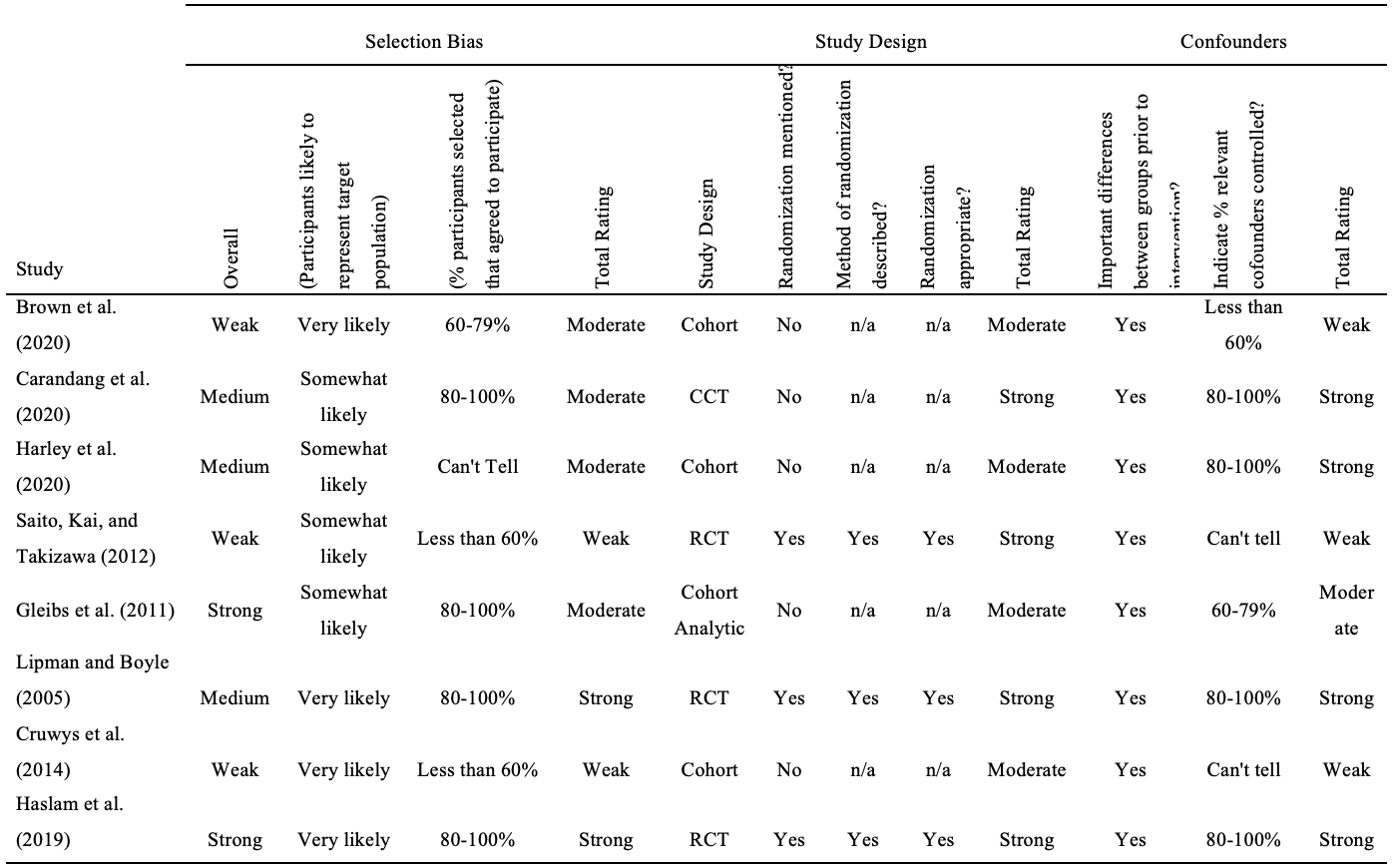
Full Quality Evaluation Criteria table of the final included studies utilising the Effective Public Health Practice Project (EPHPP) quality assessment tool (Thomas et al., 2004).


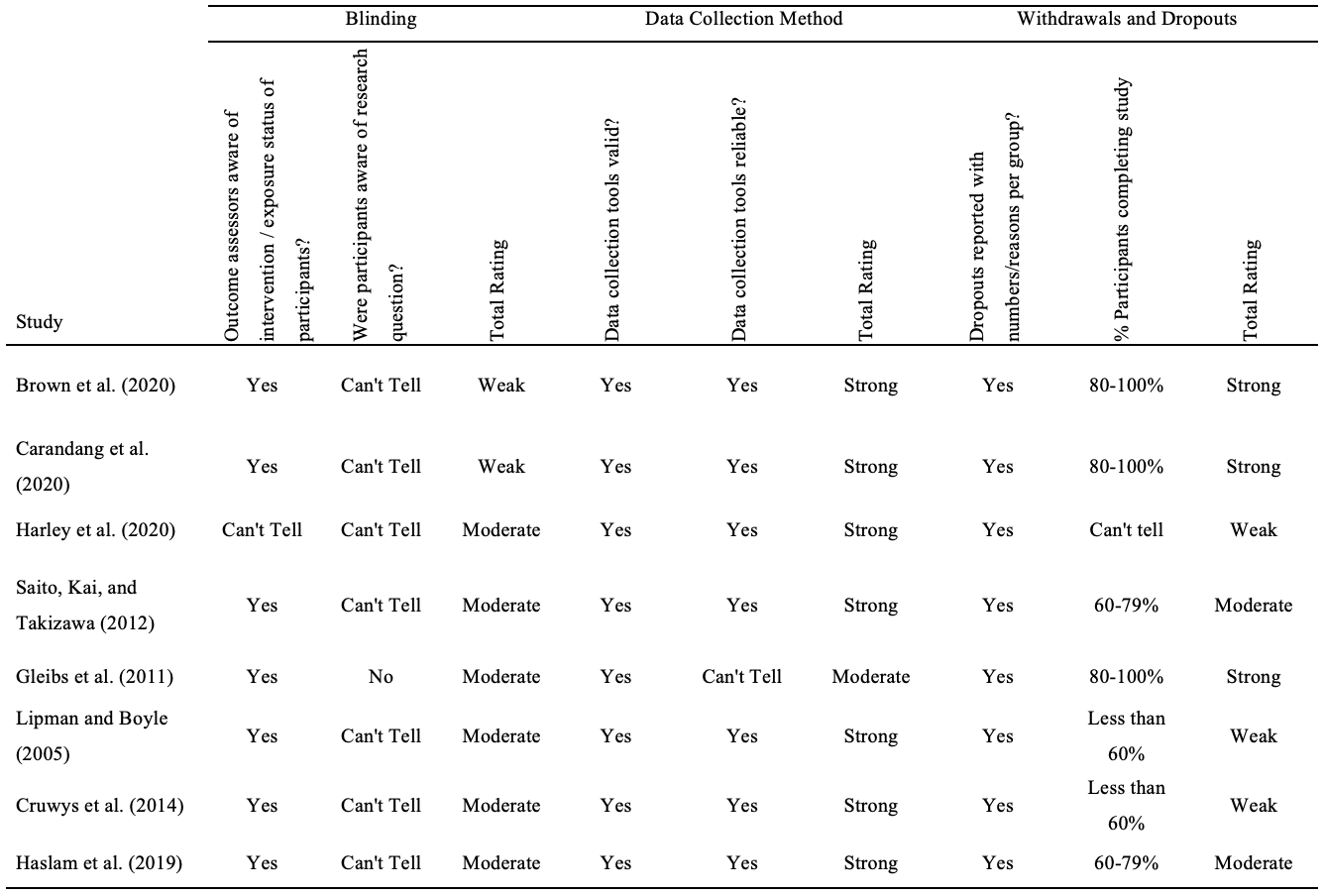


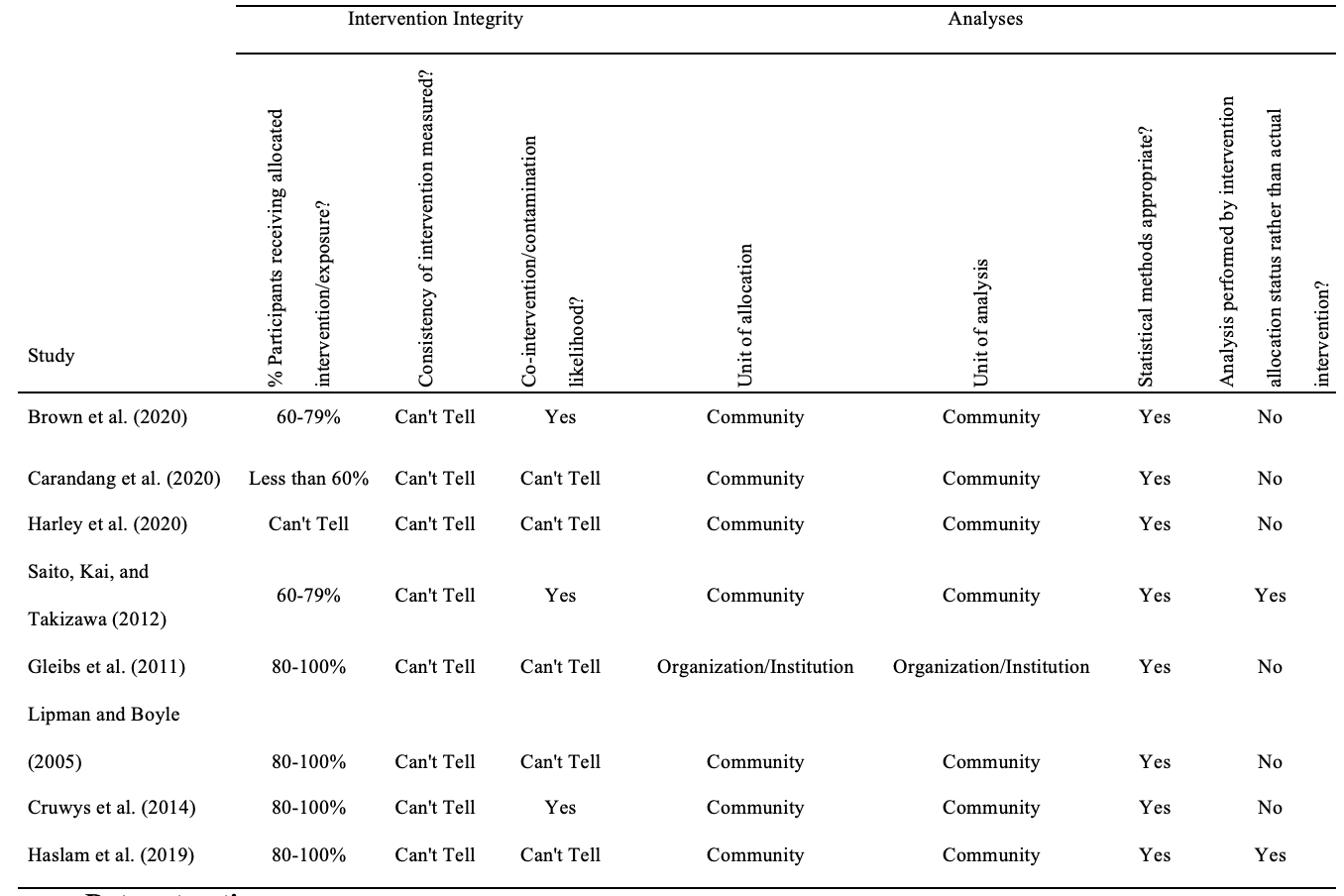


Search strategy example based on OVID search guidelines

(*formal or *structured or *official) AND

(community or (friendship AND group) or (peer AND group) or (support AND group) or (social

AND (connect* or capital or group or belonging* or relationship* or member*) or belonging or connect* or member*) AND

(community AND ((mental AND health) or wellbeing or (mental AND wellbeing) or (social AND wellbeing) or depression or anxiety or (depression AND anxiety)) AND adj3 (outcome* or intervention*))


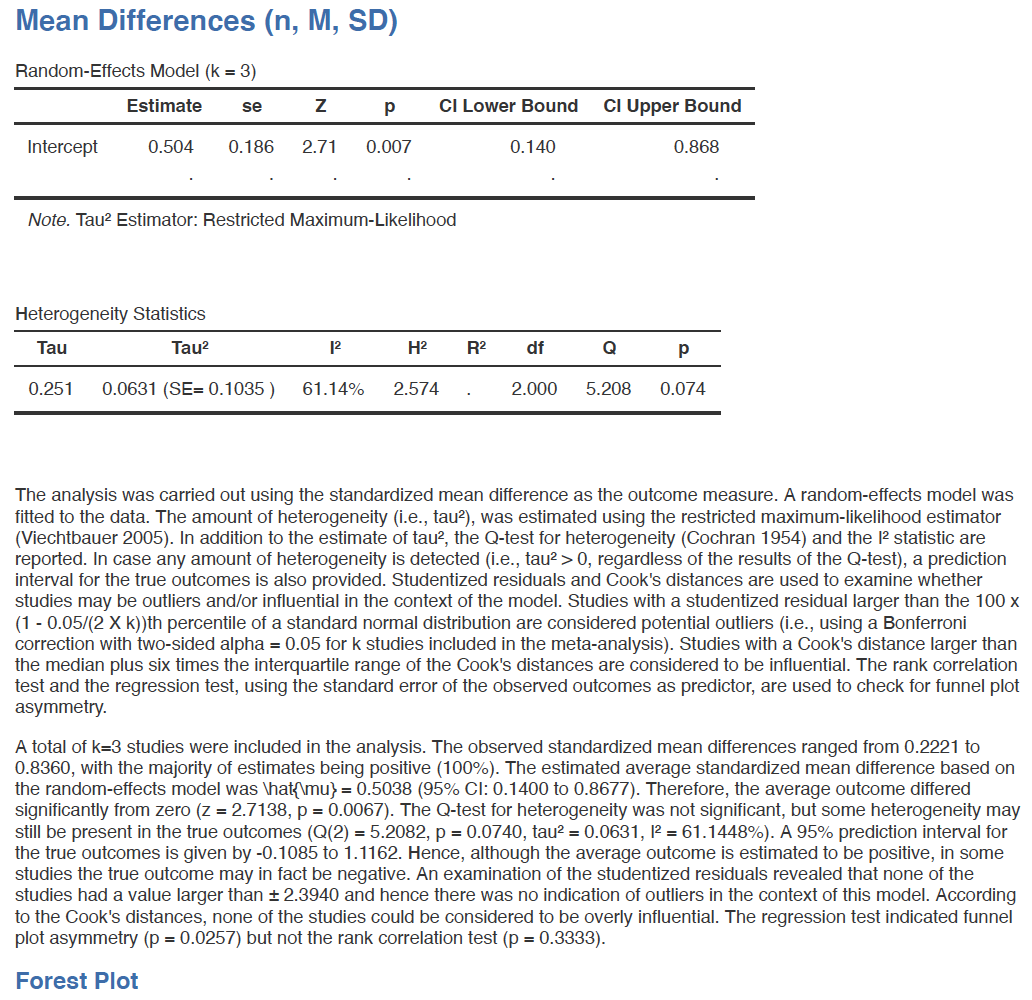
Analysis output for initial random-effects meta-analysis for depression outcomes prior to removal of Carandang et al. (2020); conducted via Jamovi.


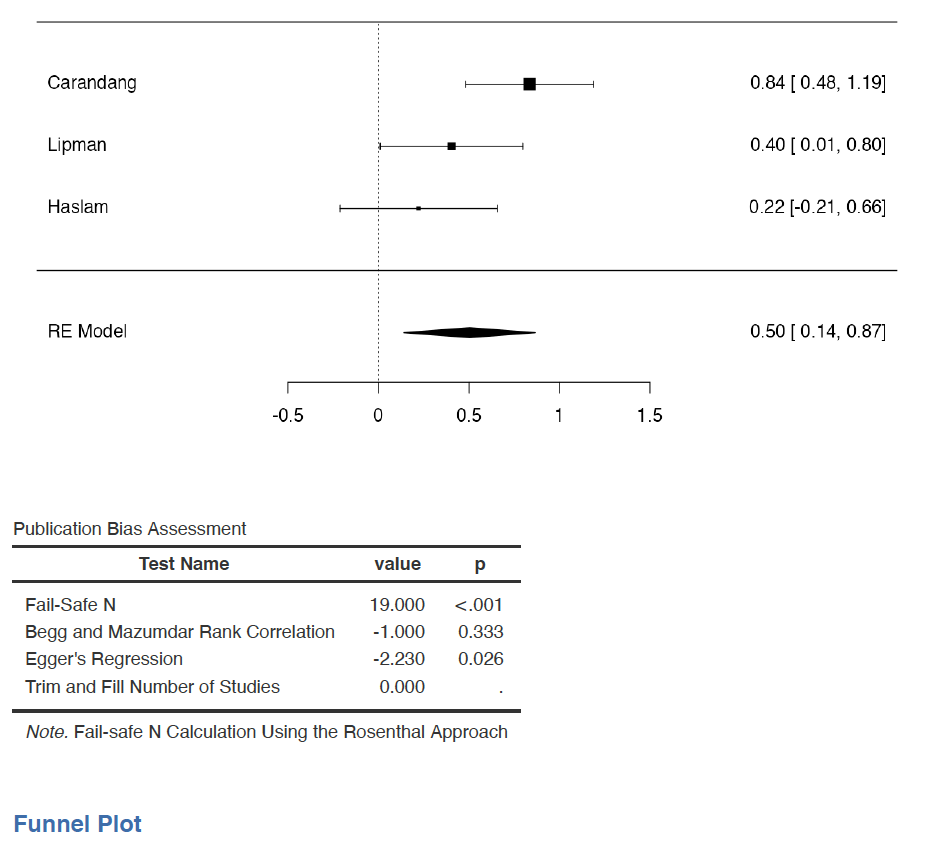

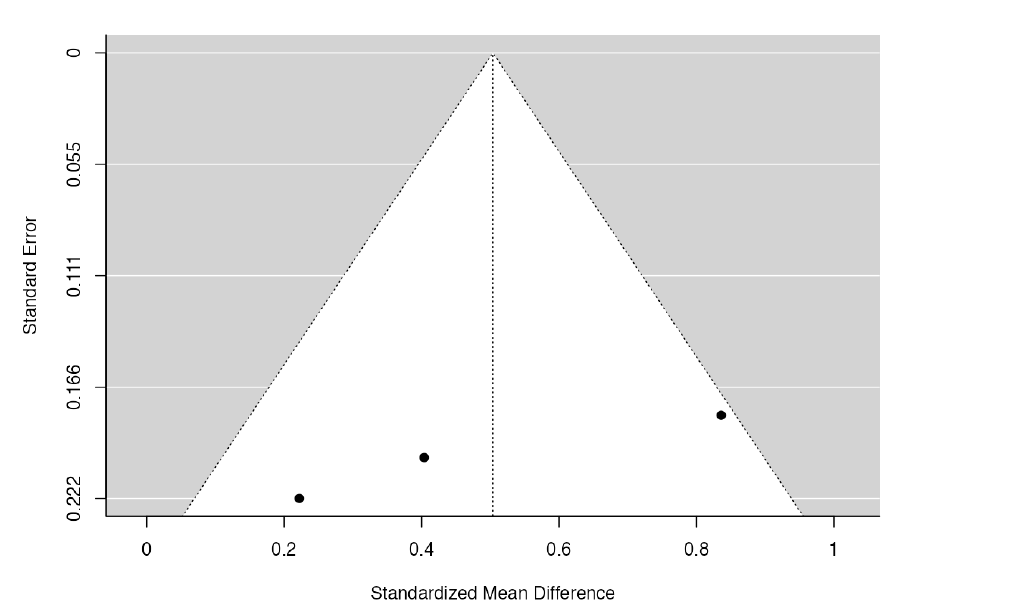


List of studies and exclusion reasons at full-text screening stage prior to final list of included studies determined.

| **Wrong intervention** | **Meta-analyses/Reviews** |
| --- | --- |
| Aschbrenner, K. A., Naslund, J. A., & Bartels, S. J. (2016, 2016--). A mixed methods study of peer-to-peer support in a group-based lifestyle intervention for adults with serious mental illness. *Psychiatric rehabilitation journal, 39*(4), 328-334. | Webber, M., & Fendt-Newlin, M. (2017, 2017--). A review of social participation interventions for people with mental health problems. *Social psychiatry and psychiatric epidemiology, 52*(4), 369-380. |
| Field, T., Diego, M., Delgado, J., & Medina, L. (2013, 2013--). Yoga and social support reduce prenatal depression, anxiety and cortisol. *Journal of bodywork and movement therapies, 17*(4), 397-403. | Nagy, E., & Moore, S. (2017, 2017--). Social interventions: An effective approach to reduce adult depression? *Journal of affective disorders, 218*, 131-152. |
| Gee, K. A., Hawes, V., & Cox, N. A. (2019, 2019--). Blue Notes: Using Songwriting to Improve Student Mental Health and Wellbeing. A Pilot Randomised Controlled Trial. *Frontiers in psychology, 10*(101550902), 423. | Dingle, G. A., Sharman, L. S., Haslam, C., Donald, M., Turner, C., Partanen, R., Lynch, J., Draper, G., & van Driel, M. L. (2021, 2021--). The effects of social group interventions for depression: Systematic review. *Journal of affective disorders, 281*, 67-81. |
| Goodman, M. L., Elliott, A. J., Gitari, S., Keiser, P., Onwuegbuchu, E., Michael, N., & Seidel, S. (2020, 2020--). Come Together to Decrease Depression: Women's mental health, social capital, and participation in a Kenyan combined microfinance program. *The International Journal of Social Psychiatry*, 20764020966014. | E, O., S, H., R, V. d. B., & O, D. (2020, 2020). A systematic review of intimate partner violence interventions focused on improving social support and/ mental health outcomes of survivors. PLOS ONE, 15(6), e0235177. https://pubmed.ncbi.nlm.nih.gov/32584910/ |
| Gustafsson, S., Berglund, H., Faronbi, J., Barenfeld, E., & Ottenvall Hammar, I. (2017, 2017--). Minor positive effects of health-promoting senior meetings for older community-dwelling persons on loneliness, social network, and social support. *Clinical interventions in aging, 12*(101273480), 1867-1877. | Lee, C., Kuhn, I., McGrath, M., Remes, O., Cowan, A., Duncan, F., Baskin, C., Oliver, E. J., Osborn, D. P. J., Dykxhoorn, J., Kaner, E., Walters, K., Kirkbride, J., Gnani, S., Lafortune, L., & Programme, N. S. P. M. H. (2021, 2021--). A systematic scoping review of community-based interventions for the prevention of mental ill-health and the promotion of mental health in older adults in the UK. *Health & social care in the community*(9306359). |
| Henteleff, A., & Wall, H. (2018, 2018). The HANS KAI Project: a community-based approach to improving health and well-being through peer support. *Le projet HANS KAI : une approche communautaire visant a ameliorer la sante et le bien-etre grace au soutien par les pairs., 38*(3), 135-146. | M, N., H, M., D, G., G, S., S, L., & F, S. (2019, 2019-10-21). A Scoping Review and Conceptual Model of Social Participation and Mental Health among Refugees and Asylum Seekers. International Journal of Environmental Research and Public Health, 16(20). https://pubmed.ncbi.nlm.nih.gov/31640210/ |
| Ho, A. P. Y. (2007, 2007). A peer counselling program for the elderly with depression living in the community. *Aging & Mental Health, 11*(1), 69-74. | Newlin, M., Webber, M., Morris, D., & Howarth, S. (2015, 2015). Social Participation Interventions for Adults with Mental Health Problems: A Review and Narrative Synthesis. Social Work Research, 39(3), 167. https://link.library.curtin.edu.au/gw?url=https://www.proquest.com/scholarly-journals/social-participation-interventions-adults-with/docview/1707087429/se-2?accountid=10382 |
| McIinnis-Perry, G. J., & Good, J. M. (2006, 2006). A psychoeducational codependency support group for older adults who reside in the community: friends supporting friends. *Journal of gerontological nursing, 32*(8), 32-42. | Reagon, C., Gale, N., Enright, S., Mann, M., & van Deursen, R. (2016, 2016--). A mixed-method systematic review to investigate the effect of group singing on health related quality of life. *Complementary therapies in medicine, 27*(9308777), 1-11. |
| Lindsay-Smith, G., O'Sullivan, G., Eime, R., Harvey, J., & van Uffelen, J. G. Z. (2018, 2018--). A mixed methods case study exploring the impact of membership of a multi-activity, multicentre community group on social wellbeing of older adults. *BMC Geriatrics, 18*(1), 226. | Ronzi, S., Orton, L., Pope, D., Valtorta, N. K., & Bruce, N. G. (2018, 2018--). What is the impact on health and wellbeing of interventions that foster respect and social inclusion in community-residing older adults? A systematic review of quantitative and qualitative studies. *Systematic reviews, 7*(1), 26. |
| Oyama, H., Watanabe, N., Ono, Y., Sakashita, T., Takenoshita, Y., Taguchi, M., Takizawa, T., Miura, R., & Kumagai, K. (2005, 2005). Community-based suicide prevention through group activity for the elderly successfully reduced the high suicide rate for females. *Psychiatry and clinical neurosciences, 59*(3), 337-344. | Nguyen, T., Holton, S., Thach, T., & Fisher, J. (2019, 2019). Informal mental health interventions for people with severe mental illness in low and lower middle-income countries: A systematic review of effectiveness. The International Journal of Social Psychiatry, 65(3), 194-206. https://link.library.curtin.edu.au/gw?url=https://www.proquest.com/scholarly-journals/informal-mental-health-interventions-people-with/docview/2222581082/se-2?accountid=10382 |
| PM, P., T, H., J, B., G, P., LA, M., JR, H., JC, K., CH, W., & JD, P. (2008, 2008-11). Can social capital be intentionally generated? a randomized trial from rural South Africa. Social science & medicine (1982), 67(10), 1559-1570. https://pubmed.ncbi.nlm.nih.gov/18771833/ |  |
| M., S., D., C., K., M., & S., A. (2001, 2001). Promoting Positive Affect and Diminishing Loneliness of Widowed Seniors Through a Support Intervention. *Public Health Nursing, 18*(1), 54-63. |  |
| Zhang, C., Zhao, H., Zhu, R., Lu, J., Hou, L., Yang, X. Y., Yin, M., & Yang, T. (2019, 2019). Improvement of social support in empty-nest elderly: results from an intervention study based on the Self-Mutual-Group model. *Journal of public health (Oxford, England), 41*(4), 830-839. |  |
| Boen, H., Dalgard, O. S., Johansen, R., & Nord, E. (2012, 2012--). A randomized controlled trial of a senior centre group programme for increasing social support and preventing depression in elderly people living at home in Norway. *BMC Geriatrics, 12*(100968548), 20. |  |
| Total: 14 | Total: 10 |

Table continued.

| **No/wrong Social outcome included** | **Wrong population** | | **Wrong study design** | **Study Protocol missed** |
| --- | --- | --- | --- | --- |
| Fleming, A. S., Klein, E., & Corter, C. (1992, 1992). The effects of a social support group on depression, maternal attitudes and behavior in new mothers. *Journal of child psychology and psychiatry, and allied disciplines, 33*(4), 685-698. | | Boda, Z., Elmer, T., Voros, A., & Stadtfeld, C. (2020, 2020--). Short-term and long-term effects of a social network intervention on friendships among university students. *Scientific reports, 10*(1), 2889. | Mawani, F. N., & Ibrahim, S. (2021, 2021--). Building Roads Together: a peer-led, community-based walking and rolling peer support program for inclusion and mental health. *Canadian journal of public health = Revue canadienne de sante publique, 112*(1), 142-151. | Page-Reeves, J., Murray-Krezan, C., Regino, L., Perez, J., Bleecker, M., Perez, D., Wagner, B., Tigert, S., Bearer, E. L., & Willging, C. E. (2021, 2021--). A randomized control trial to test a peer support group approach for reducing social isolation and depression among female Mexican immigrants. *BMC Public Health, 21*(1), 119. |
| J., S., M., J., A., N., G., K., S., M., Westbrook J.I. AO - Siette, J., & http://orcid.org/---, O. (2021, 2021). A mixed-methods study evaluating the impact of an excursion-based social group on quality of life of older adults. *BMC Geriatrics, 21*(1), 356. http://www.biomedcentral.com/bmcgeriatr/ | |  | Jones, M., Kimberlee, R., Deave, T., & Evans, S. (2013, 2013--). The role of community centre-based arts, leisure and social activities in promoting adult well-being and healthy lifestyles. *International Journal of Environmental Research and Public Health, 10*(5), 1948-1962. |  |
| Total: 2 | | Total: 1 | Total: 2 | Total: 1 |
